# Cryogenic and Dissolution DNP NMR on γ-Irradiated Organic Molecules

**DOI:** 10.1021/jacs.4c04041

**Published:** 2024-07-19

**Authors:** Angeliki Giannoulis, Korin Butbul, Raanan Carmieli, Jihyun Kim, Elton Tadeu Montrazi, Kawarpal Singh, Lucio Frydman

**Affiliations:** † Department of Chemical and Biological Physics, Weizmann Institute of Science, 234 Herzl Street, Rehovot 7610001, Israel; ‡ Department of Chemical Research Support, Weizmann Institute of Science, 234 Herzl Street, Rehovot 7610001, Israel; § Department of Chemistry Education, Kyungpook National University, Daegu 41566, Republic of Korea; ∥ Yusuf Hamied Department of Chemistry, University of Cambridge, Cambridge CB2 1EW, U.K.

## Abstract

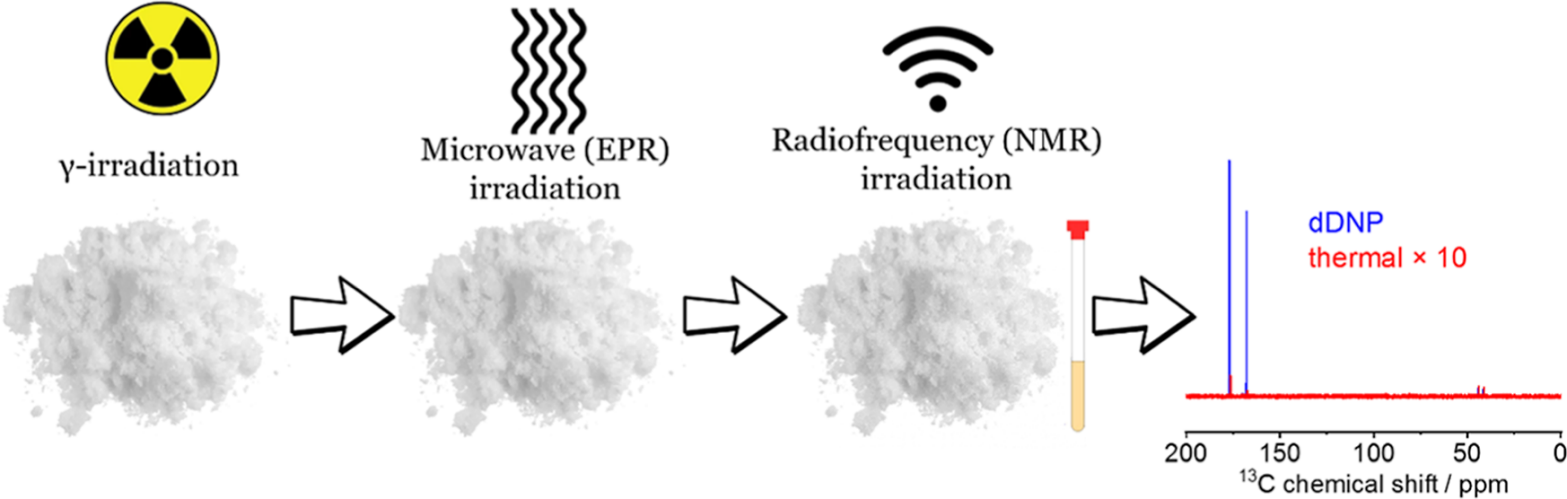

Nuclear magnetic resonance (NMR) plays a central role in the elucidation of chemical structures but is often limited by low sensitivity. Dissolution dynamic nuclear polarization (dDNP) emerges as a transformative methodology for both solution-state NMR and metabolic NMR imaging, which could overcome this limitation. Typically, dDNP relies on combining a stable radical with the analyte within a uniform glass under cryogenic conditions. The electron polarization is then transferred through microwave irradiation to the nuclei. The present study explores the use of radicals introduced via γ-irradiation, as bearers of the electron spins that will enhance ^1^H or ^13^C nuclides. ^1^H solid-state NMR spectra of γ-irradiated powders at 1–5 K revealed, upon microwave irradiation, signal enhancements that, in general, were higher than those achieved through conventional glass-based DNP. Transfer of these samples to a solution-state NMR spectrometer via a rapid dissolution driven by a superheated water provided significant enhancements of solution-state ^1^H NMR signals. Enhancements of ^13^C signals in the γ-irradiated solids were more modest, as a combined consequence of a low radical concentration and of the dilute concentration of ^13^C in the natural abundant samples examined. Nevertheless, ca. 700–800-fold enhancements in ^13^C solution NMR spectra of certain sites recorded at 11.7 T could still be achieved. A total disappearance of the radicals upon performing a dDNP-like aqueous dissolution and a high stability of the samples were found. Overall, the study showcases the advantages and limitations of γ-irradiated radicals as candidates for advancing spectroscopic dDNP-enhanced NMR.

## Introduction

Dynamic nuclear polarization (DNP) has emerged as a powerful complement to nuclear magnetic resonance (NMR), enhancing the sensitivity of this spectroscopy by exploiting the higher intrinsic magnetic moments of electronic spins.^1−3^ This sensitivity enhancement is achieved via a microwave (mw) irradiation in the proximity of the electron paramagnetic resonance (EPR) line of a polarizing agent comixed with the sample of interest, which drives a transfer of spin alignment from the electrons to the surrounding nuclei.^4,5^ Although carried occasionally in solutions at high fields,^6−9^ this process is most efficient when implemented on glassy cryogenic solids, where the polarizing electrons are uniformly distributed and EPR relaxation times are sufficiently long to support mw saturation.^10,11^ Polarization on electron-proximate nuclei will then be enhanced via a number of mechanisms,^5,9,12^ in a process that will be subsequently propagated throughout the material by spin diffusion.^13^ Thanks to their high natural abundance and high gyromagnetic ratio, protons are both the predominant acceptors of DNP and effectors of this spin-diffusion. For most chemical and biophysical DNP applications, stable organic radicals such as nitroxides, phenyl-allyls, or trityls are commonly introduced exogenously as polarizing agents, often by coimmersing them with the target molecules in a glassing solvent that when frozen acts as a polarizing matrix.^14−16^ The carbon-centered organic radicals, in particular, exhibit narrow EPR spectra, which can be efficiently excited by mw pulses or saturated by continuous irradiation, and favorable relaxation properties for performing DNP under cryogenic conditions. In addition to codissolving these polarizing agents in a glassy matrix, the material of interest can also be coated with a polarizing agent solution via an incipient wetness impregnation,^10,13^ or “swelled”^17,18^ by a solution containing the polarizing agent.

An alternative way for the homogeneous introduction of radicals within the bulk of a solid material involves the application of radiation—including UV-based,^19,20^ γ-rays,^21,22^ or neutron radiation.^23,24^ Unlike impregnation, where radicals may predominantly reside on the surface of the crystals, these methodologies can generate radicals within the core of a polycrystalline material. This could facilitate the electron-to-nucleus polarization transfer, while a homogeneous distribution of the generated radicals could favor subsequent spin diffusion processes. Additionally, radiation-based implantations could facilitate the study of systems of limited solubility^11^ or prone to solvent-induced phase transitions;^25^ radiation-derived radicals could also exhibit advantages in a subsequent dissolution DNP (dDNP) process.^22,26,27^ Indeed, as demonstrated for UV-irradiated samples,^20^ introducing radicals in such a way minimizes the sample’s pre-DNP dilution, and prolongs the post-DNP nuclear spin relaxation (*T*_1_) by the spontaneous quenching of the unstable radiation-derived radicals brought about by the dissolution solvent. Similar advantages have been demonstrated when radicals were generated in bulk via electrical discharges.^28−30^ In the specific case hereby considered of stable radicals generated by γ-irradiation, Rossini and co-workers demonstrated the generation of suitable internal polarizing centers in this manner for inorganic materials such as quartz, whose solid-state ^29^Si NMR could be enhanced at room temperature,^31^ as well as for the magic angle spinning (MAS) NMR of γ-irradiated organic solids at 105 K.^32^ This work explores the use of γ-irradiation as generated by a cobalt-60 source, to induce radical formation in powder samples, and the feasibility of exploiting the ensuing samples for performing dDNP. We found that γ-irradiation enabled the performance of DNP in numerous—but not all—organic compounds explored, as revealed by ^1^H NMR signal enhancements in the solid state at cryogenic temperatures. Sudden dissolution of these powders in water showed significant enhancements of the solution-state ^1^H NMR signals. Repeating the same procedure on directly polarized ^13^C nuclei delivered only moderate enhancements for the solution-state ^13^C NMR signals of quaternary and methylene groups. This limited performance is ascribed to the relatively low concentration of the radicals achieved by the irradiation process, coupled to the low efficiency of ^13^C spin-diffusion under natural abundance conditions. Approaches to overcome this limitation and further extensions of these capabilities are briefly discussed.

## Experimental Section

### Sample Preparation and Radical Formation

To generate radicals in the bulk of solids, a series of organic compounds were sent in two main batches to Sorvan Ltd. (Yavne, Israel), a γ-irradiation facility specializing in the sterilization of pharmaceuticals, cosmetics, and food products. Batch no. 1 included 0.08 g of Ala, GlyGly, glucose, and sucrose (Ala = l-alanine, Gly = glycine), which were evacuated and flame-sealed in their respective NMR tubes before being exposed to irradiation doses of 150 kGy. Batch no. 2 comprised 0.05 g of AlaAla, AlaAsp, GlyAla, Gly methylester HCl, ATP, succinic acid, sodium pyruvate, ibuprofen, salicylic acid, and dl-4-fluorophenylAla (Asp = aspartic acid, ATP = adenosine triphosphate), which were exposed to ambient oxygen levels and irradiated with a lower dose of 50 kGy. Results (see below) confirmed that these differences in irradiation doses and in the presence/absence of oxygen did not affect significantly the radicals’ concentrations; thereafter, several of the above-mentioned compounds were sent independently to the irradiation facility and subjected to varying irradiation doses (50–100 kGy) for the purposes of subsequent experiments. Table S1 summarizes the different samples used in different experiments. All of these samples, as well as BDPA and 4-aminoTEMPO radicals, were purchased from Sigma-Aldrich and used without further purification. Radicals generated by γ-irradiation were found to remain stable and delivering steady DNP enhancements for several months (cf. Figure S1), and all the irradiated materials were tested to be nonradioactive before the MR measurements. Ancillary measurements were done using Ox063 radicals purchased from Oxford Instruments, as well as using ^13^C_1_-pyruvic acid and U-^13^C_6_-glucose obtained from CortecNet. A batch of U-^13^C_6_-glucose was also sent to γ-irradiation for the sake of assessing the ^13^C-driven spin diffusion effects.

### Continuous Wave EPR, DNP, and NMR Measurements

Room-temperature continuous-wave EPR (CW-EPR) spectra were recorded on a Bruker ELEXSYS E500 X-band (∼9.4 GHz) spectrometer using ∼1 mg of samples packed in 1.0 mm inner diameter capillaries made of clear fused quartz and closed at both ends with Critoseal. Spin counting was done using the XEPR software, and experimental parameters are given in the figures’ legends. Echo-detected EPR (ED-EPR) spectra were recorded at 5 K on a home-built hybrid pulsed-EPR-NMR spectrometer^33^ at a magnetic field of 3.38 T, corresponding to a ^1^H Larmor frequency of ∼143 MHz and an electron Larmor frequency of ∼95 GHz. The spectra were recorded using the α–τ–α–τ–echo sequence with microwave pulses of α = 300 ns and echo delay time of τ = 600 ns and integrating the echo at half width while sweeping the magnetic field. This spectrometer’s sample cup was made of Teflon to minimize the ^1^H background signal and accommodated ∼30 mg of the sample.

Solid-state ^1^H DNP enhancements were monitored by collecting ^1^H NMR signals at ∼143 MHz utilizing a customized solid-state probe suitable to fit on a Hypersense (HS) Oxford Instruments polarizer operating at ∼94 GHz electron Larmor frequencies and 1.5 K temperatures.^34^ The solid NMR coil was mounted on a Kel-F holder to minimize ^1^H background signals, and accommodated a saddle 6 mm diameter coil geometry. A Kel-F sample container fitting ∼70 mg of the sample was screwed in firmly into this coil-holder for the measurements. Microwaves were generated and controlled by the HS polarizer, and unless otherwise stated, they were applied at 150 mW nominal powers. ^1^H NMR spectra were acquired using a Varian/Agilent console. Each spectrum was collected as a single scan using a presaturation train on the ^1^H achieved by applying 100 × 100 μs-long radiofrequency (rf) pulses, followed by a DNP polarization time and concluded with an rf pulse (typically 4 μs, ca. 10°) and a 0.128 ms acquisition time. ^13^C signals of the irradiated samples were also measured at 1.5 K, but this time using the built-in internal ^13^C coil that the HS brings for measuring its polarization build-up, tuned at ∼36 MHz. Prior to these build-up measurements a presaturation on the ^13^C was achieved by 3 × 250 μs-long pulses, followed by a 14 μs rf pulse (corresponding to ca. 5°). These data were processed by the standard Hypersense software RINMR. Solid-state ^1^H DNP enhancements were also measured on the home-built hybrid spectrometer used for collecting ED-EPR spectra^33^ at 5 K. These ^1^H spectra were recorded using a presaturation 50 × 15 μs-long pulse train, followed by a DNP pumping process and concluded with a 90° pulse (typically 6–8 μs) and a 0.512 ms acquisition time. All solid-state NMR data were processed by applying 3000 Hz line broadening, zero filling, and Fourier transform. The data from HS were treated in absolute mode and the area of each spectrum was used, whereas spectra from the hybrid pulsed-EPR-NMR were phase-corrected, and the ensuing peak intensity used for the analysis. All ^1^H NMR data were processed with a home-written Matlab script. Enhancement factor calculations also required the acquisition of NMR data without DNP as well as of the empty sample containers; these measurements were also preceded by suitable presaturation pulses.

Solution-state ^1^H and ^13^C spectra were collected at 330 K on a 11.7 T (500 MHz ^1^H frequency) Magnex magnet equipped with a Bruker AvanceNeo console and a Bruker Prodigy cryo-probe. NMR experimental parameters were as follows: ^1^H NMR data were collected as a train of single-scan FIDs separated by a 50 ms recycle delay, each with an acquisition time of 1 s, a receiver gain of 1, and an excitation pulse length of 0.5 μs, corresponding to a ca. 5° pulse. As often postdissolution ^1^H signals were too intense and saturated the probe’s cold preamplifier, the latter was bypassed (via a software command) when performing the ^1^H acquisitions. ^13^C NMR involved a similar train of single-scan FIDs with an acquisition time of 1 s, receiver gain of 2, relaxation delay of 0.05 s, pulse length of 2 μs (corresponding to ca. 10°), and ^1^H decoupling applied with a garp4 modulation at 8.9 W powers. All data were analyzed using TopSpin 4.1.4 and home-written Matlab scripts.

## Results

### EPR Features of the Irradiated Samples

**Figure 1:**
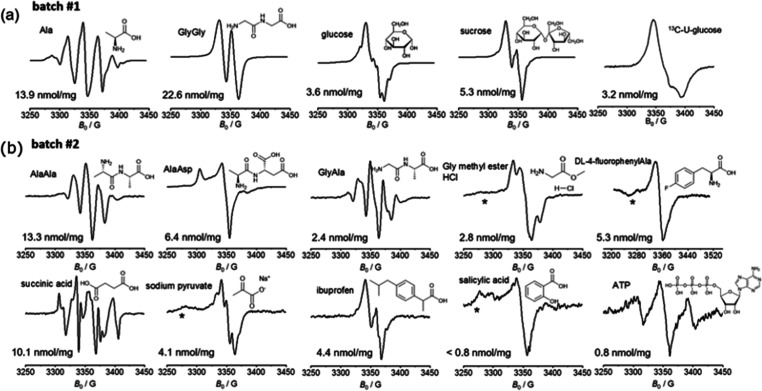
Room-temperature X-band (9.4 GHz) CW-EPR spectra of γ-irradiated powders measured in the solid state. The structures of the samples sent to irradiation are given along with the electron spin concentration per milligram of powder estimated by spin counting. Some of the spectra display a background signal from the resonator marked with an asterisk (*). CW-EPR conditions: attenuation 10 dB (AlaAsp, Gly methylester HCl, succinic acid, sodium pyruvate, ibuprofen, salicylic acid, dl-4-fluorophenylAla), or 25 dB (Ala, GlyGly, glucose, sucrose, AlaAla, GlyAla), conversion time 40 ms, modulation amplitude 1 G, modulation frequency 100 kHz, 1 scan.

The room-temperature X-band EPR spectra in Figure 1 confirm the generation of radicals for various γ-irradiated compounds, with radical concentrations varying between 0.8 and 23 nmol per mg of sample. The main mechanism for radical generation via γ-irradiation in solids is the direct ionization process, where γ-photons ionize molecules by ejecting electrons from their outer orbitals, leading to the formation of radical cations and free electrons.^35^ The nature of the resulting radicals depends on factors such as chemical structure, irradiation dose, and temperature; for example, aromatic compounds produce less radicals due to the resonance stabilization of the radical resulting in excitation instead of ionization;^35^ this is consistent with results we observed for salicylic acid and ATP. Regarding the remaining samples: γ-irradiation of Ala is known to yield two main radical species (R1, R2), with R1 being a carbon-centered radical where the amide group has been abstracted, and R2 is the radical after abstraction of a hydrogen from the central carbon of the Ala molecule. Additionally, a minor species R3, where the radical is delocalized from the nitrogen of the amide group to the carbonyl oxygen, has also been identified.^36^ This complexity is reflected in the CW-EPR spectrum of Ala. AlaAla and GlyAla yield similar spectra, suggesting the presence of similar radical species; additionally, the CW-EPR spectrum of GlyAla is similar to the one reported in the literature for AlaGly,^37^ suggesting that the order of amino acids is not critical for the nature of the radical species in these dipeptides. The radical of GlyGly has been assigned to the NH_2_ĊHCONHCH_2_COOH species after abstraction of a hydrogen from the methylene group, consistent with its CW-EPR spectrum.^37^ The most complicated EPR spectrum is that of succinic acid, which was found to form HOOCĊHCH_2_COOH radical species upon γ-ray irradiation.^38^ Glucose was found to form two carbon-centered radical species upon γ-irradiation, one of the form RĊHOH, product of the hydrogen abstraction, and one on the C3 of the 6-membered ring.^32^ The radical of sucrose has been proposed to be on the 6-membered ring.^39^ The spectra of salicylic acid and dl-4-fluorophenylAla were broad and featureless, in agreement with previous observations.^32^ The CW-EPR spectra of these compounds are not previously reported but resemble those of γ-irradiated aspirin (acetylsalicylic acid),^40^ where three different radical species were identified, and of paracetamol [*N*-(4-hydroxyphenyl)acetamide],^41^ where two different radical species were found. In the case of aspirin, these are of the form RCOȮ, RĊH_2_ and a third due to hydrogen addition at one of the carbons in the ring,^40^ whereas for paracetamol, one radical was found to be carbon-based while the other is a hydroxyl radical after breakdown of C–OH bonds. When compared to samples belonging to “batch no. 1″, the smaller irradiation doses and exposure to oxygen that characterized powders in batch no. 2 do not, collectively, reflect significantly lower radical concentrations. Additionally, when a given compound was sent for γ-irradiation under vacuum and exposed to varying doses in the 50–150 kGy range, no significant radical concentration differences were observed upon testing (Figure S1a). Further, most radicals were found to be stable at ambient conditions over several weeks or months (Figure S1b), and some are being used for DNP studies even 2 years after their γ-irradiation. Dissolving the powders in H_2_O resulted in the rapid disappearance of the EPR signal (Figure S1c), confirming that the radicals thus produced are not stable in aqueous solutions—a potentially important feature for subsequent dissolution DNP experiments.

### Solid-State Cryogenic DNP NMR

**Figure 2:**
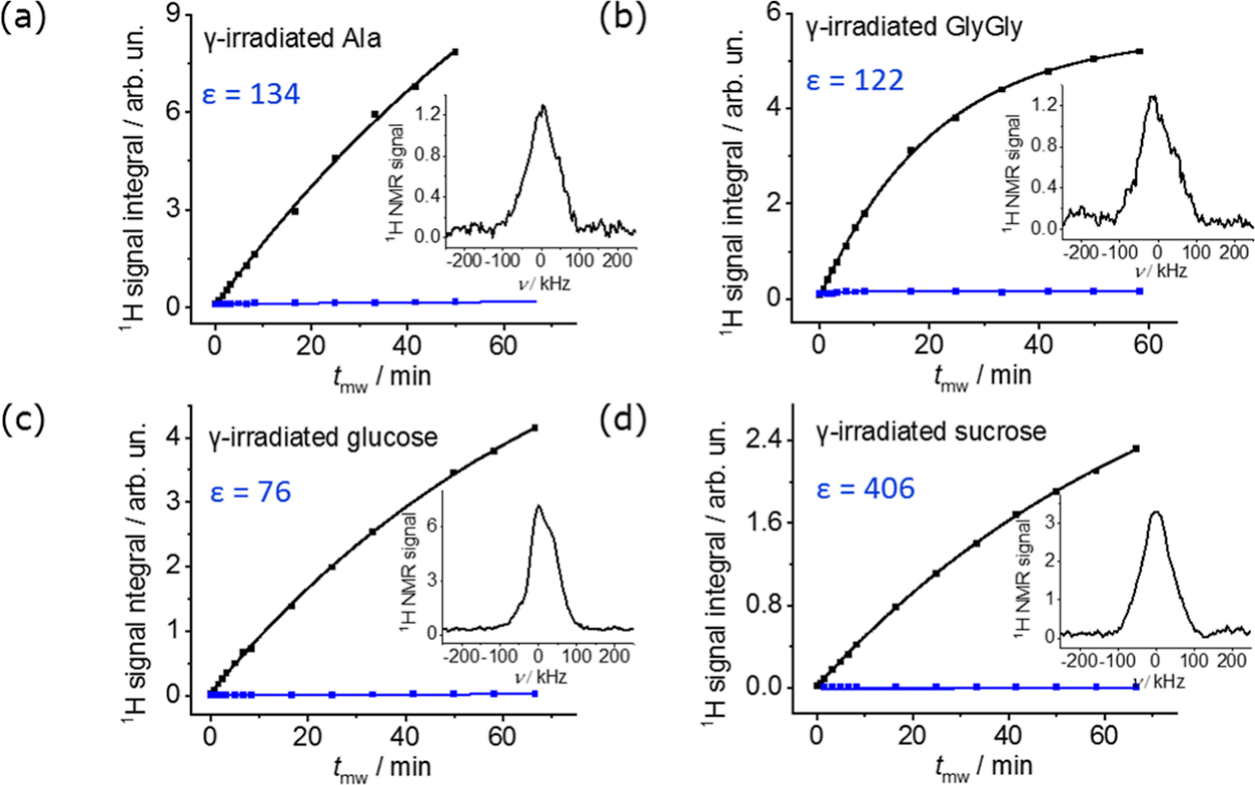
^1^H signals vs mw irradiation times of the γ-irradiated powders from “batch no. 1” in the solid state, measured at 1.5 K on a Hypersense polarizer (∼94 GHz) using a home-built ^1^H coil tuned at ∼143 MHz. ^1^H signal buildups were measured in the presence and absence of microwaves (black and blue points, respectively) as described in the Experimental Section. The solid black and blue lines are fits of the data to single exponentials, from which enhancements were calculated by dividing *A*_DNP_ and *A*_thermal_ values after correcting for the ^1^H signal background of an empty sample cup [see text; mw off data in (d) fell on a straight line whose end value was taken as the *A*_thermal_ signal value]. Given in the insets are representative solid-state ^1^H NMR spectra recorded with 4 μs pulse lengths and 100 s DNP polarization times (*t*_mw_).

Figure 2 presents ^1^H NMR signals of γ-irradiated powders belonging to “batch no. 1” as a function of magnetization buildup times in both the presence and absence of microwaves, as recorded at 1.5 K on a HS polarizer. Spins in these buildup runs were initially presaturated, and their recovering signal amplitudes *A* were then fitted to single exponentials as
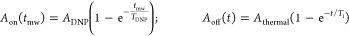
where *t*_mw_ is the microwave irradiation time in the DNP experiments, *t* is the postsaturation time elapsed in the microwave off experiment, and *T*_DNP_ and *T*_1_ are the build-up times in the presence and absence of microwave irradiation, respectively. From these data, the long-term signal amplitudes *A*_DNP_ and *A*_thermal_ were estimated. As at the same temperature, an empty sample cup was found to have a ^1^H signal *A*_bkgrnd_ contributing to a residual background (Figure S2a), *A*_bkgrnd_ values were subtracted from the sample’s signal amplitude in both the presence and absence of microwaves. From all these data and corresponding fits, background-corrected DNP enhancement values  were calculated.

*A* values, along with corresponding build-up times and effective enhancements ε, are given in Table 1. All samples included in batch no. 1 gave robust ^1^H NMR enhancements ≥75. Similar measurements on salicylic acid from batch no. 2 did not afford any measurable DNP (Figure S2c); this can be attributed to the small amount of radical present in this sample postirradiation (see Figure 1). Assuming that at 3.35 T and 1.5 K, the samples have a thermal polarization (*P*_thermal_) of 8 × 10^–4^, then the percent DNP polarization (% *P*_DNP_) can be calculated from these enhancements as % *P*_DNP_ = ε × 8 × 10^–2^ (Table 1). Even though the buildup is slow (ca. 20–90 min depending on sample), the overall polarization (% *P* > 6% at all cases) is higher than that achieved for a 10 mM 4-aminoTEMPO solution in H_2_O/glycerol (3/2) (Figure S3): *P* ≈ 4.3% at 1.5 K (see also ref (42)). We also measured for this sample a buildup time of ca. 1 min (Table 1), a fast buildup vis-à-vis the irradiated samples that we attribute to a higher radical concentration.

**Table 1:** Buildup Parameters of Various γ-Irradiated Samples as Derived from Fitting the ^1^H NMR Data Measured at 1.5 or 5 K (in Non-Bold or Bold, Respectively) to a Single Exponential Component (Unless Otherwise Stated)

	microwave status	amplitude (*A*)/arb. un.	polarization buildup time (*T*_DNP_, *T*_1_)/min	^1^H enhancement (ε) in solid state	% *P*_DNP_ at 1.5 K	^1^H enhancement (ε) in the liquid state for nonexchangeable hydrogens at 11.7 T
γ-irradiated Ala 150 kGy	on	186	92	134	11	460
	off	1.4	91			
γ-irradiated GlyGly 150 kGy	on	55.7	21	122	9.8	720
	off	0.5	4			
γ-irradiated glucose 150 kGy	on	710	77	76	6.1	
	off	9.3	322^a^			
γ-irradiated sucrose 150 kGy	on	397	77	406	33	
	off	0.98^b^	n.a.^b^			
25 mM TEMPO in 3/2 H_2_O/glycerol^c^			10^c^		3.9^c^	
10 mM 4-aminoTEMPO in 3/2 H_2_O/glycerol	on	7.6	1.3	54	4.3	
	off	0.14	3.2			
						
**γ-irradiated Ala 50 kGy**	**on**	**42^d^**	**0.8**	**137^d^^,^^b^**	**11^d^^,^^b^**	
	**off**	**0.31^d^^,^^b^**	**n.a.**			
**γ-irradiated AlaAla 50 kGy**	**on**	**1.8^d^**	**0.6**	**72^d^**	**5.8^d^**	**200**
	**off**	**0.25^d^**	**0.5**			
**γ-irradiated ATP 150 kGy**	**on**	**14.8^d^**	**2.6**	**28^d^**	**2.3^d^**	
	**off**	**0.5^d^**	**2.0**			
**γ-irradiated sucrose 50 kGy**	**on**	**89^d^**	**2.0**	**100^d^**	**8.0^d^**	
	**off**	**0.89^d^**	4.5			
**γ-irradiated GlyGly 50 kGy**	**on**	**89.5^d^**	**1.7**	**>190^e^**	**>15^e^**	
	**off**	**0.47^d^^,^^e^**	**2.3**			
**γ-irradiated glucose 100 kGy**	**on**	**161^d^**	**2.0**	**224**	**18**	
	off	0.7^d^	7.4			

a The buildup time of the microwave off measurements was too long to be accurately determined with the number of collected data points.

b The intensities of the microwave off data were too low to provide a reliable fit of buildup time *T*_1_. This sample was measured again (Figure S2b) using slightly less microwave power (100 mW) and an enhancement of >300 was again found.

c From ref (42), the ε value refers to microwave on data only (i.e., it is just *A*_DNP_).

d Data collected on a hybrid NMR/EPR/DNP W-band spectrometer at 5 K.

e The microwave off data could not be corrected for the signal of the empty cup; therefore, only a lower limit can be given for ε and % *P* (only the microwave on data were corrected for the signal of the empty cup).

**Figure 3:**
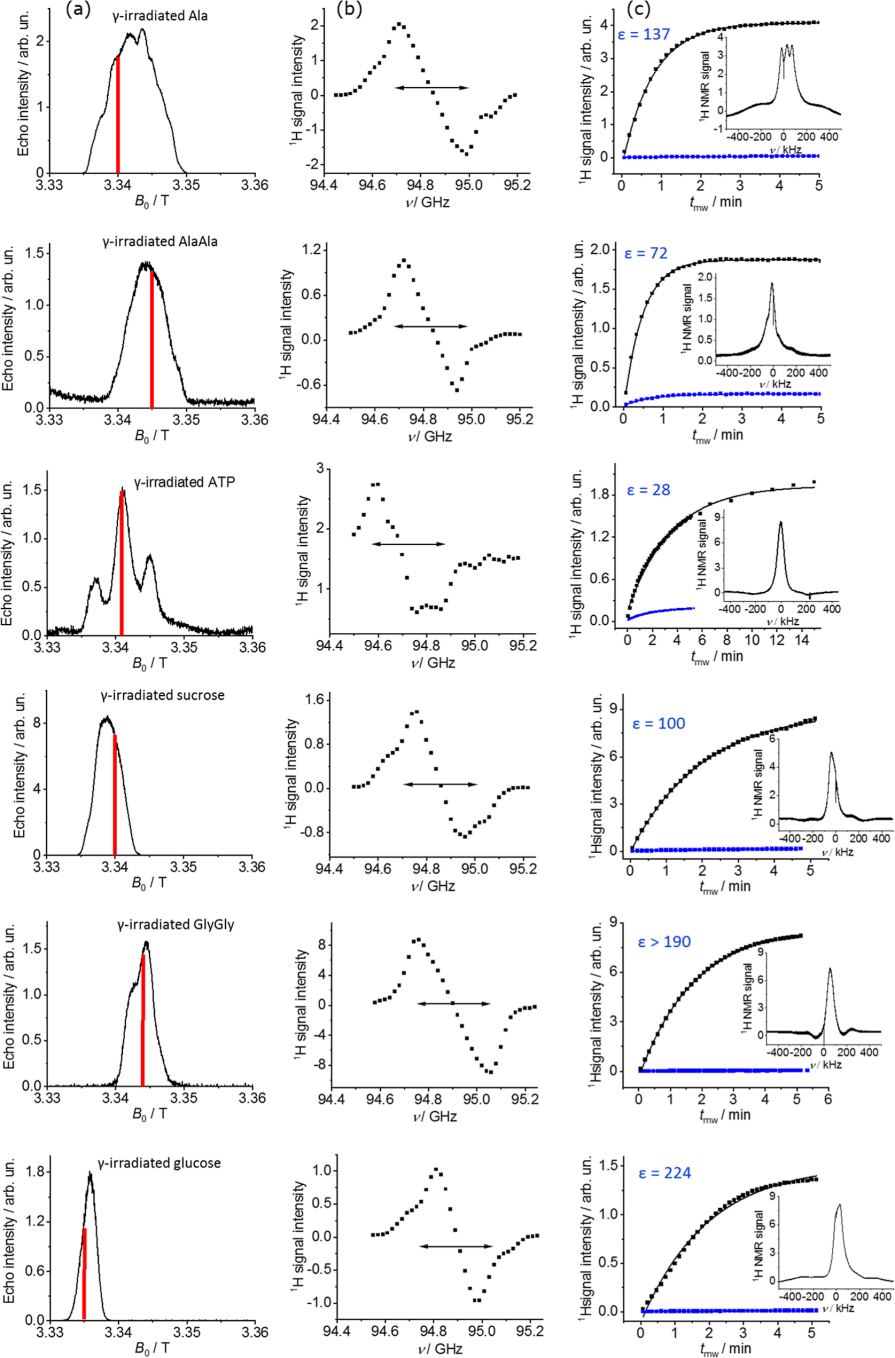
EPR and DNP results arising for γ-irradiated samples (Ala, AlaAla, sucrose, and GlyGly after 50 kGy irradiation, ATP at 150 kGy, glucose at 100 kGy) in the solid state at 5 K. (a) Echo-detected W-band (∼95 GHz) EPR spectra. (b) ^1^H NMR DNP enhancement profiles recorded with 20 s polarization time and 6 μs (for Ala, ATP, glucose) or 8 μs (for AlaAla, sucrose, GlyGly) 90° pulses, with arrows denoting the 286 MHz frequency separation reflecting twice the ^1^H Larmor frequency at this field. (c) ^1^H signals vs mw irradiation times in the presence and absence of microwaves (black and blue points, respectively) with the solid lines being the fit to the data to single exponentials. Enhancement were calculated as described in the text. The red lines in (a) denote the position where microwave pulses were applied for monitoring the ^1^H polarization buildup shown in (c).

To further characterize these samples and identify the mechanism driving these ^1^H polarization buildups, the frequency profiles of the electron → nuclear polarization transfers were investigated. We attempted to measure this for the γ-irradiated samples by observing the ^1^H signal enhancement vs mw frequency at 1.5 K; however, given our Hypersense’s limited mw frequency sweeping bandwidth, the entire frequency profile for the various radicals could not be resolved using this setup. Still, in those cases where radicals were sufficiently narrow, polarization profiles typical of the solid effect characterizing organic radicals could be identified (Figure S4). To further confirm this, measurements were made on a home-built EPR/NMR/DNP W-band spectrometer that,^33,43^ although operating at slightly higher temperatures, allowed us to both collect EPR spectra and scan DNP enhancements over sufficiently large frequency ranges. Figure 3 shows results obtained on this setup on various γ-irradiated powdered samples at 5 K, including echo-detected EPR (ED-EPR) spectra, along with their ^1^H DNP frequency profiles and the NMR signal buildups in the presence and absence of microwaves at the same temperature. The ED-EPR spectra (Figure 3a) were distinct in terms of their line shape and widths, with Ala showing the largest full width at half-maximum (fwhm) at 223 MHz and glucose the narrowest (68 MHz). Examination of these lineshapes also revealed fine structures; ATP, for instance, exhibits an EPR spectrum characterized by three distinct peaks. These fine structures can be attributed to hyperfine couplings (Figure S5a)—in the ATP case arising from either protons or the naturally abundant ^14^N in the purine basis. As for their DNP enhancement profiles (Figure 3b), most of the irradiated samples show a frequency separation of their positive and negative lobes in the ∼240–290 MHz range. This is ca. twice the ^1^H Larmor frequency at W-band (∼143 MHz) and indicative of a solid effect mechanism.^44^ However, the DNP frequency profiles of some samples—ATP in particular—are more complicated than simple Gaussian lineshapes sited at the frequencies expected from a typical solid effect, probably witnessing again the effects of electron radicals with multiple hyperfine couplings. One can, however, exclude the possibility of significant cross effects^5^ or thermal mixing mechanisms^45^ in the observed enhancements, as these would result in narrower profiles not evidenced even for relatively sharp EPR line widths like that of glucose. Neither do the data support a significant Overhauser contribution for these DNP profiles,^46,47^ as these would exhibit a characteristic enhancement on-resonance with the EPR peak.

In terms of DNP performance, these 5 K ^1^H NMR data were again fitted to monoexponential functions in both the presence and absence of microwaves (Figure 3c). After correcting for the background signal of an empty cup (vide infra), all irradiated samples afforded similar DNP enhancements at 5 K as those obtained at 1.5 K using the Hypersense polarizer (Table 1), suggesting similar underlying DNP mechanisms. Additional tests on 150 kGy-irradiated glucose and 150 kGy-irradiated Ala samples corresponding to different batches gave enhancements similar to those reported in Figure 3, confirming a robust DNP performance under different sample preparations or irradiation doses (Figure S6). Still, the buildup times at 5 K were ca. an order-of-magnitude faster than those measured at 1.5 K. This could reflect the shorter *T*_1_ relaxation times expected upon increasing several-fold the absolute temperatures (Table 1). Another feature that could explain both the similar ^1^H DNP performances and the different buildup times observed in the Hypersense and home-built spectrometers used here relates to the higher mw powers available in the latter. Even though the nominal power in the hybrid spectrometer was not explicitly measured for each sample, tests showed that powers of ∼770 mW were, in principle, achieved using this setup; this compares with the ∼150 mW powers employed on the Hypersense. Additionally, a trend emerged between the ED-EPR spectral line widths and the enhancements observed, in the sense that narrower EPR lines yielded larger enhancements. This was particularly evident for the case of glucose, which has the narrowest EPR line and affords the largest ε of 224 at this temperature. ATP, on the other hand, with a complex, wide EPR line, afforded the smallest enhancement when placing the microwaves on its central peak. Moving the microwaves to the right or left hyperfine peaks afforded only the background signal from the cup (Figure S5b).

**Figure 4:**
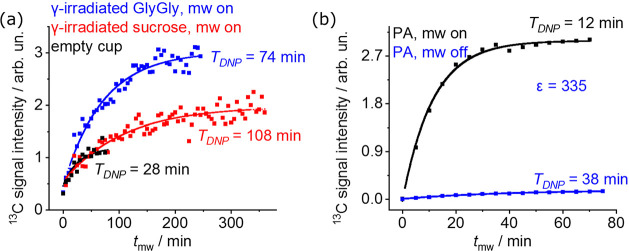
^13^C signals vs buildup times of (a) γ-irradiated powders and (b) 100 μL of ^13^C_1_-pyruvic acid (PA) containing 15.4 mM Ox063 trityl radical in the solid state (∼1.5 K)—both measured on a HS polarizer (∼94 GHz) using the internal ^13^C coil tuned at ∼36 MHz. In (a), ^13^C signals were measured in the presence of microwaves (blue and red points for GlyGly and sucrose, respectively) having as reference the corresponding measurement for an empty sample cup (black color), while in (b) the sample was measured in the presence (black) and absence (blue) of microwaves. For all measurements a 5° pulse length was used. The solid lines are fits of the data to single exponentials, from which the given buildup times were estimated. Microwave conditions: (a) 177 mW and 93.945 GHz for GlyGly, 183 mW and 94.045 GHz for sucrose, and 100 mW and 94.05 GHz for the empty sample cup, (b) 0 mW (mw off) or 100 mW (mw on), 94.05 GHz. In (b), ε was calculated as *A*_DNP_/*A*_thermal_, as found from the fits.

Similar measurements were performed on the γ-irradiated samples but monitoring the buildup of the ^13^C signals at 1.5 K using the Hypersense polarizer. We found that the signal intensity of the ^13^C nucleus was 2–3 orders magnitude lower compared to that of ^1^H and only marginally higher than the corresponding of the empty sample cup (Figure 4a). This is expected, given the low natural abundance (∼1.1%) of ^13^C in all the samples sent to γ-irradiation. Furthermore, the signals built up slowly—in time scales that were similar to those observed for the corresponding ^1^H measurements at the same temperature. For comparison, the buildup time and overall enhancement of a ^13^C-enriched sample—^13^C_1_-pyruvic acid comixed with 15.4 mM Ox063 trityl radical as the polarizing agent—were ε = 335 and *T*_DNP_ = 12 min (Figure 4b). The slower buildup times of the γ-irradiated samples can be attributed to the slowness of the spin-diffusion process among the dilute ^13^C reservoirs—even if the relatively wide DNP profiles evidenced in the ^1^H-detected experiments might also mean that the similar polarization time scales evidence a ^1^H-driven buildup of the ^13^C nuclei. For completion, we recorded similar measurements on a γ-irradiated sample of uniformly ^13^C_6_-enriched glucose (vide infra).

### Dissolution DNP ^1^H NMR

**Figure 5:**
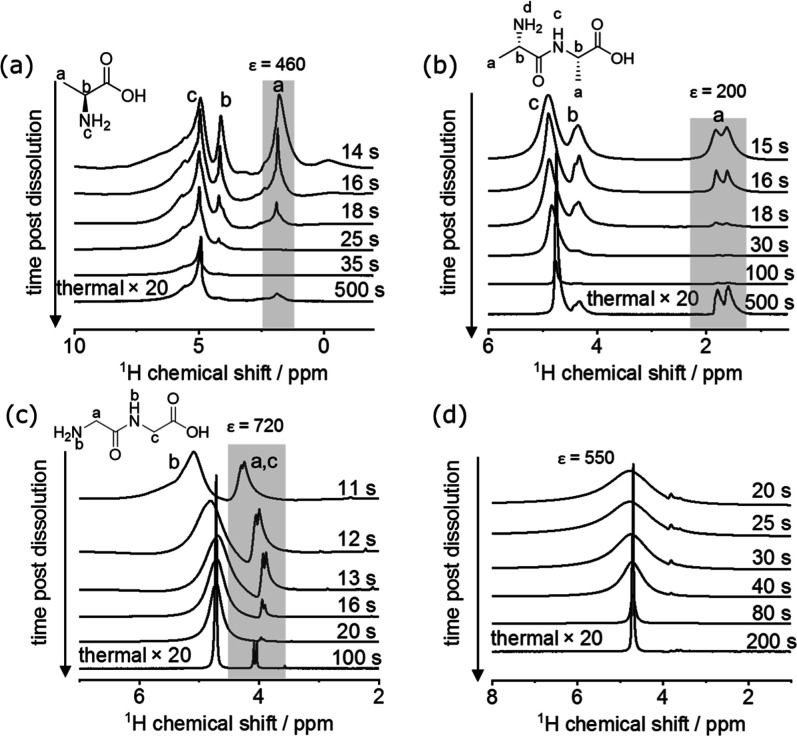
dDNP ^1^H NMR results on γ-irradiated (a) Ala (150 kGy), (b) AlaAla (50 kGy), (c) GlyGly (150 kGy), and (d) water/glycerol (8/2) monitoring ^1^H chemical shifts. 50 mg (AlaAla) or 100 mg (Ala, GlyGly) of γ-irradiated powders and 75 μL of 4-aminoTEMPO 20 mM was polarized on a HS polarizer at 1–1.5 K and 93.9 GHz, 170 mW, 3 h polarization time for Ala, and AlaAla, at 93.945 GHz, 177 mW, 5.5 h polarization time for GlyGly and 94.125 GHz, 150 mW, 1 h for 4-aminoTEMPO. Dissolutions were done with 3.5 mL of D_2_O and ^1^H solution state NMR spectra were recorded on a 500 MHz ^1^H frequency spectrometer at 330 K using a 1 s acquisition time, 0.05 s relaxation delay, 0.5 μs pulse length (corresponding to ca. 5°) for irradiated samples or 0.8 μs for 4-aminoTEMPO, 1 scan. The data were recorded as a pseudo-2D with the post-dissolution time being the pseudo dimension and a time interval of 1 s from spectrum to spectrum. Representative spectra at various times were selected. Insets give the structures analyzed with the various protons indicated and chemical shifts assigned to the spectra. The chemical shifts of CH_3_ (for Ala, AlaAla) or CH_2_ (for GlyGly) groups that were used for estimating the ^1^H NMR signal enhancements are highlighted with a gray background.

Having characterized DNP enhancements in the solid state, the in situ performance of γ-irradiated radicals on ^1^H signal enhancements in solutions following a dissolution DNP process was also tested.^48^ To do so, 50–100 mg samples of γ-irradiated powders were placed in a Peek cup, introduced in the HS magnet, and subjected to microwave irradiation over suitable durations (usually hours) at 1–1.5 K. With the ^1^H ensemble thus hyperpolarized, 3.5 mL of superheated D_2_O were flushed at ca. 10 bar into the cryogenic hyperpolarized pellet via plastic tubing, and the melted solution transferred onward with a stream of helium gas to a nearby liquid state 500 MHz NMR spectrometer. Figure 5 shows some of the postdissolution ^1^H NMR spectral series arising then for Ala, AlaAla, and GlyGly. For all three compounds, hyperpolarized ^1^H signals are observable from the nonexchangeable hydrogens, even if spectra immediately after dissolution are shifted and broadened due to radiation damping.^49^ This behavior is similar to that reported in previous observations.^42,50^ The ^1^H NMR spectra of the labile NH and NH_2_ groups in these molecules are broadened due to exchanges with the hydrogens from HDO; this broadening becomes severe due to the hyperpolarization of the amines/amides in Ala, AlaAla, and GlyGly. Eventually, these broadened peaks disappear, and their protons end up contributing to a single, thermally polarized, HDO resonance. Spectra are not presented for glucose and sucrose, as due to these sugars’ substantial number of exchangeable protons, these samples served as excellent sources for creating postdissolution hyperpolarized water by exchanges with the D_2_O—while the proximity of the nonexchangeable sugar resonances to the signal arising from this hyperpolarized water prevented their latter observation. Unfortunately, due to solubility issues, H_2_O was the sole solvent that could instantly dissolve all these compounds for rapidly performing the dDNP experiment; use of other solvents such as acetonitrile was assayed, but these could not dissolve these polar powders. Solubility seemed to proceed more readily when similar compounds were dissolved from glassing solutions (data not shown), which might aid in a more complete solubilization, albeit at the expense of diluting the targeted samples.

Following the samples’ sudden dissolution, ^1^H NMR signals became narrower and less intense over time, primarily due to loss of polarization dictated by the ^1^H *T*_1_ relaxation (excitation pulses used to interrogate the signal were small, see the Experimental Section). Given the multiple peaks in the NMR spectra and their shifting positions, it is convenient to characterize these decays by considering the first point of the signals’ FIDs (equivalent to the full area under the corresponding NMR spectra) as a function of postinjection time. For Ala and AlaAla, fitting in such way the FID profile with an exponential function afforded 2.2 ± 0.01 and 5.8 ± 0.02 s lifetimes, respectively (Figure S7a,b). Interestingly, the profile of signal decay for AlaAla could not be fitted to a monoexponential decay despite multiple dissolution trials, something that we attribute to the presence of particles that were not instantly dissolved during the sudden dissolution/flushing process. This is supported by the line widths of the nonexchangeable protons at thermal equilibrium, which did not yield narrow lines as expected for homogeneous solutions. Similar broadenings at thermal equilibrium were observed for Ala, again suggesting the presence of particles not being dissolved by the flushing. This happened despite performing the dissolution of the irradiated Ala powders with D_2_O adjusted to pD 3.0 to facilitate the amine protonation; dissolution on Ala samples at pD 7.0 gave spectra affected by radiation damping effects (characteristic signal increase upon quenching of radiation damping effects, see Figure S7c), due to the slowness of the amine ⇔ water proton exchange process at neutral pH. Similar fits for irradiated GlyGly with a monoexponential function afford 13.9 ± 0.2 s postdissolution life times (Figure S7d). This ^1^H signal decay is slower than that reported for arginine hyperpolarized in a solution containing 25 mM TEMPO (decay time ≈10.9 ± 0.1 s^42^) or for water protons when hyperpolarized using TEMPO^42,51^ or 4-hydroxyTEMPO^51^ (∼4 s). GlyGly’s slower decay may be reflecting the quench of the implanted radicals upon water dissolution. Upon reaching thermal equilibrium, the H_2_O peak at 4.7 ppm ends up dominating the single-shot NMR spectra of all compounds. Still, quantification of the signal enhancement afforded by these dissolution experiments can be done by measuring the signal intensity immediately after dissolution and at thermal equilibrium. To account for the different line shapes between polarized and nonpolarized spectra, we corrected the signal intensities while taking into account differences in the line broadening of the hyperpolarized and thermal peaks and found ε of 460, 200, and 720 for the nonexchangeable protons—CH_3_ in Ala and AlaAla, and CH_2_ in GlyGly, respectively. (This approach based on peak heights and widths was found more reliable than using signal integrals for quantifying the enhancements, as in some cases the early postdissolution spectra showed phase distortions that prevented dependable area calculations). Similar experiments on arginine hyperpolarized by 25 mM TEMPO gave enhancement ε = 438,^42^ while H_2_O and DMSO protons hyperpolarized by 25 mM TEMPO or 4-hydroxyTEMPO afforded enhancements of ε ≈ 500.^51^ Additionally, we performed here a reference measurement using 20 mM 4-aminoTEMPO hyperpolarized for 1 h and found a water enhancement of 550 under conditions similar to our samples (Figures 5d and S7e). It follows that for achieving ^1^H DNP enhancement, the γ-irradiated radicals work as good or better than externally mixed nitroxide radicals.

### ^13^C Dissolution DNP NMR

**Figure 6:**
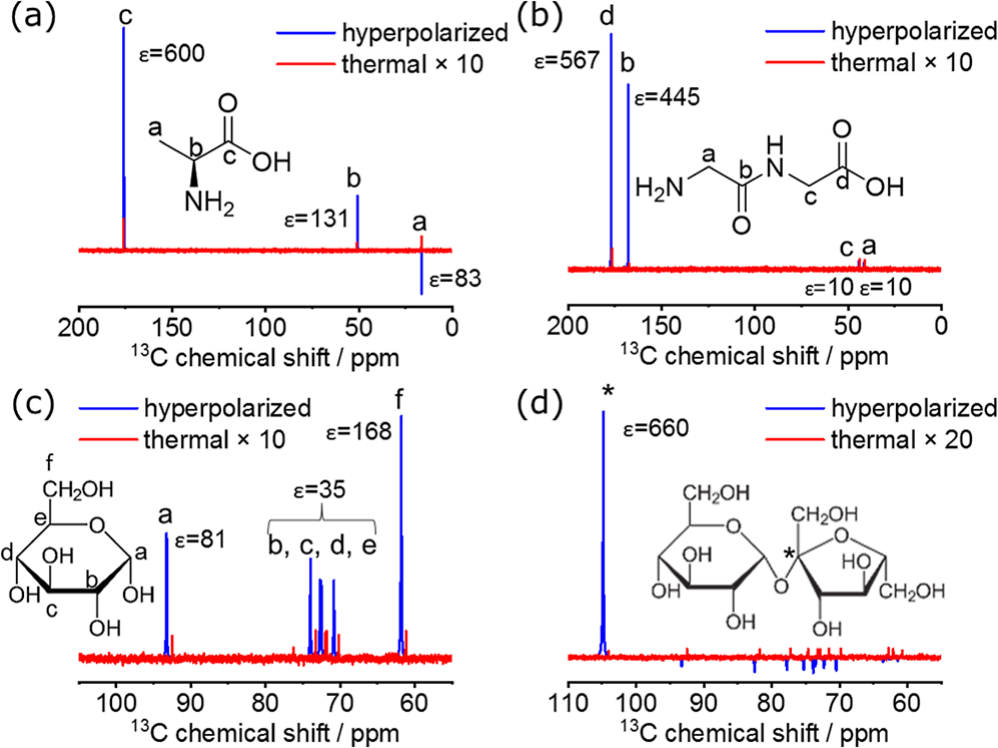
^13^C solution state dDNP NMR results on 150 kGy γ-irradiated (a) Ala, (b) GlyGly, (c) glucose, and (d) sucrose. 100 mg of the γ-irradiated powders was polarized on a HS polarizer at 1–1.5 K and at 94.045 GHz using 183 mW of microwave power; 5.5 h polarization times were used for Ala, 4 h for GlyGly and glucose, and 8.5 h for sucrose. Dissolutions were done with 3.5 mL of H_2_O/D_2_O (9/1) and spectra were recorded on a 500 MHz ^1^H frequency spectrometer at 330 K using 1 s acquisition time, 0.05 s relaxation delay, 2 μs (≈13°) pulse length, 1 scan. The data were recorded as pseudo-2D with the time being the pseudo dimension and a time interval of 1 s from spectrum to spectrum. Hyperpolarized (immediately postdissolution) vs thermal (FID at 100 s) spectra are shown in blue and red colors, respectively. For calculating the enhancements, a thermal spectrum was recorded with 100 scans after the end of the dissolution process using a long relaxation delay. Given in the insets are the structure of the powders with the various carbons indicated and the chemical shifts assigned directly on the spectrum along with the ε values. For sucrose, the quaternary carbon is indicated with an asterisk.

Although the expectations from the solid-state data (Figure 4) predict that low levels of hyperpolarization will arise from direct DNP of natural abundance ^13^C species, dissolution experiments were still performed to monitor the ^13^C solution NMR signals of the irradiated powders after dDNP (Figure 6). As in the ^1^H NMR experiments above, 100 mg of the irradiated powders were subjected to microwave irradiation over several hours at 1–1.5 K, before being flushed with 3.5 mL of H_2_O/D_2_O (9/1) via a plastic tubing into a close-by liquid state 500 MHz NMR spectrometer. 10% D_2_O allowed locking of the sample in the NMR magnet after dissolution, and ^1^H decoupling (80 μs garp decoupling pulses) was used to simplify the spectra. Here, the enhancement was calculated as the signal-to-noise ratio (SNR) of the hyperpolarized spectrum (1 scan) over the SNR of thermal multiscan spectra collected on the postdissolution samples, corrected by the number of scans. Overall, all compounds afforded relatively low enhancements, with the carbonyl carbons in Ala and GlyGly, as well as the quaternary carbon in sucrose exhibiting the largest ε of 500–700, due to their longer *T*_1_. The ^13^C signal enhancements obtained for these γ-irradiated samples are similar to those seen for radicals induced by electrical discharges (ε ∼ 700).^27^ The overall time evolution of the signal collected after dissolution (FID profiles in Figure S8) shows ^13^C *T*_1_ values \ comparable to that of ^1^H (ranging ∼6–13 s)—apart from the sites in glucose—which were found shorter. The rates of signal decay for the various ^13^C sites are in general agreement with the observed enhancements for each site: the slower the relaxation, the higher the enhancement. The low ε observed in these experiments—by comparison, model dissolutions on neat ^13^C_1_-pyruvic/BDPA samples were in the 12,000 (Figure S9) range in the same system—likely represent the combination of a low natural ^13^C abundance hindering good spin-diffusion, and of relatively small amounts of radicals present in these powders. Interestingly, some of the peaks appear to have an “anti-polarized” signal postdissolution (see Ala and sucrose in Figure 6). For Ala’s CH_3_ group, this probably reflects tunneling-related effects arising upon being cooled in liquid He and then suddenly melted.^52^ For sucrose, all of the carbons but the quaternary one show a similar pattern, probably reflecting cross-relaxation effects between the hyperpolarized protons and neighboring carbon-13 nuclei.^53^ To evaluate whether the low enhancements in the ^13^C NMR spectra arise from the low percent of naturally occurring ^13^C nuclei in the powders, which impedes spin diffusion, we performed similar experiments on uniformly ^13^C-labeled glucose (U-^13^C_6_-glucose) after subjecting it to γ-irradiation (150 kGy). CW-EPR confirmed the presence of radicals (Figure 1) with concentration similar to that of nonlabeled glucose. Figure S10 shows the ensuing ^13^C solid state NMR signal buildup on the Hypersense polarizer at 1.5 K with and without microwave irradiation, as well as its ^13^C NMR spectrum postdissolution. We found an enhancement in the solid state of 37, which, although larger than that observed for the natural abundance carbon-13 irradiated samples (Figure 4a), still remains an order of magnitude smaller than the enhancement corresponding to ^13^C_1_-pyruvic acid with the Ox063 radical (Figure 4b). Dissolutions on the labeled glucose yielded an average ε ≈ 800 for all the carbons. This enhancement is ca. 5× larger than that found for the nonlabeled glucose (Figure 6c), evidencing the achievement of a more uniform polarization enhancement. Still, it is ca. 4-fold smaller than what can be achieved when the same sample is polarized in an optimized preparation of 4 M U-^13^C_6_-glucose in water using 15 mM of comixed Ox063 trityl radical as polarizing agent (e.g., Figure S11).

## Conclusions

Several common organic powders were subjected to γ-irradiation to create radicals in the bulk of the materials for evaluating both liquid and solid state DNP performances on the ^1^H and ^13^C nuclei of the materials without need to dissolve these in a glassy “DNP juice”. The rationale for doing so was 2-fold: on one hand, this form of introducing the radicals could bypass the need for diluting the sample for polarizing it. In addition, although dilution would still happen during the transfer from the polarizer to the NMR magnets, the unstable nature of these radicals would lead to their rapid elimination upon subjecting them to such flushing with a hot solvent like water. In all samples and conditions tested, γ-irradiation afforded stable radicals, even if variations in the radical concentration were observed with the sample. The consistent trends observed across varying irradiation doses (Figure S1a for GlyGly and Ala and Figure S6 for glucose and Ala) suggest that a given compound is likely to perform similarly regardless of the details of the γ-irradiation. For most cases, however, radical concentrations on the 5–10 nmol/mg of the sample could be achieved; this proved sufficient for performing a competitive ^1^H DNP under cryogenic conditions. Particularly for the case of DNP-enhanced ^1^H NMR experiments (Table 1), it was found that the γ-irradiation provided better enhancements on the 1–5 K range than typical samples including externally added, comixed radicals like TEMPO. The narrow nature of the γ-derived radicals and their positioning close to other common organic radicals like BDPA also facilitated the DNP experiments. Part of this efficiency derived from the relatively low concentration of the radicals vs that normally present in a “DNP juice”, which was also reflected in relatively long ^1^H DNP buildup times. Relatively low radical concentration coupled to a low natural isotopic abundance, however, conspired against the realization of direct ^13^C DNP; arguably, the use of ^1^H → X cross-polarization while doing ^1^H-based DNP might be able to overcome this penalty.^54−56^ In general, the mechanism mediating the ^1^H DNP was identified to be the solid effect,^57,58^ although some features including the effects of hyperfine splittings remain to be elucidated. Postdissolution DNP afforded good ^1^H NMR enhancements, similar to the mixed nitroxide radicals. Issues were found regarding the presence of residual particles that were not dissolved 100% during the dissolution process and affected the line width of the postdissolution spectrum as well as exchanges with an aqueous dissolution solvent that complicated the acquisition of quality ^1^H NMR data—these, however, are likely to be complications of the dDNP experiments regardless of the radical’s origin. In fact, EPR data revealed the immediate disappearance of the γ-generated radicals upon dissolution in water, thus aiding somewhat in achieving higher enhancements and longer lifetimes in the ^1^H NMR. The postdissolution ^13^C NMR spectra were more disappointing, even if they affirmed the structural integrity of the majority of the irradiated compounds, without revealing molecular cleavage or radical-induced polymerizations.^59^ When normalizing the enhancements that γ-irradiation and electrical discharging gave on a reference sample composed of U-^13^C_6_-glucose vs optimized experiments based on polarizing the same sample with the stable Ox063 at an optimal concentration, the γ-irradiation efficiency was ≈25%, whereas the discharge-generated radicals afforded ca. 10% of the optimal enhancement. It remains to be seen how general this behavior is; it also remains to be seen whether there are additional aspects of polarizing a sample without requiring the presence of a glass; foremost, the use of ^1^H → ^13^C cross-polarization could be exploited for both ^1^H and low-γ nuclei NMR solution-state acquisitions.

## Supporting Information Available

EPR data, solid-state ^1^H NMR data, and frequency-swept DNP profiles on the Hypersense polarizer, solid-state ^1^H NMR data on the hybrid NMR-EPR spectrometer, EPR and DNP on 150 kGy-irradiated ATP, postdissolution ^1^H and ^13^C NMR FID profiles, NMR/DNP data on ^13^C_1_-pyruvic/BDPA, γ-irradiated U-^13^C_6_-glucose and U-^13^C_6_-glucose/Ox063 samples; and summary of samples used (PDF)
