# Supplementary material for: Cryogenic and Dissolution DNP NMR on γ-Irradiated Organic Molecules
Source: J Am Chem Soc. 2024 Jul 19;146(30):20758–69. doi: 10.1021/jacs.4c04041 (PMC11295201; doi:10.1021/jacs.4c04041)
Supplement: Supplementary file 1 — ja4c04041_si_001.pdf [file ja4c04041_si_001.pdf]

Supporting Information for
Cryogenic and dissolution DNP NMR on γ-irradiated organic molecules

Angeliki Giannoulis,[1,*] Korin Butbul,[1] Raanan Carmieli,[2] Jihyun Kim,[1,3] Elton Tadeu Montrazi,[1] Kawarpal Singh,[1,4] Lucio Frydman[1,*]

[1]Department of Chemical and Biological Physics, Weizmann Institute of Science, 234 Herzl St., Rehovot, 7610001, Israel

[2]Department of Chemical Research Support, Weizmann Institute of Science, 234 Herzl St., Rehovot, 7610001, Israel

[3]Department of Chemistry Education, Kyungpook National University, Daegu41566, Republic of Korea

[4]Yusuf Hamied Department of Chemistry, University of Cambridge, Cambridge CB2 1EW, United Kingdom

*corresponding authors: lucio.frydman@weizmann.ac.il, angeliki.giannoulis@weizmann.ac.il

Table of Contents

1. Additional EPR data ... S2
2. Additional solid-state ^{1}H NMR data in the Hypersense polariser ... S2
3. Frequency swept DNP profiles arising in the Hypersense polarizer ... S3
4. Additional data on 150 kGy irradiated ATP ... S4
5. Additional solid-state ^{1}H NMR data on the hybrid NMR-EPR spectrometer ... S4
6. Post-dissolution ^{1}H and ^{13}C NMR FID profiles ... S5
7. DNP/NMR data on $^{13}C_1$-pyruvic/BDPA ... S6
8. DNP/NMR data on U-$^{13}C_6$-glucose after 150 kGy γ-irradiation ... S6
9. DNP/NMR data on U-$^{13}C_6$-glucose/Ox063 ... S7
10. Summary of all samples used for DNP measurements ... S7

1. Additional EPR data

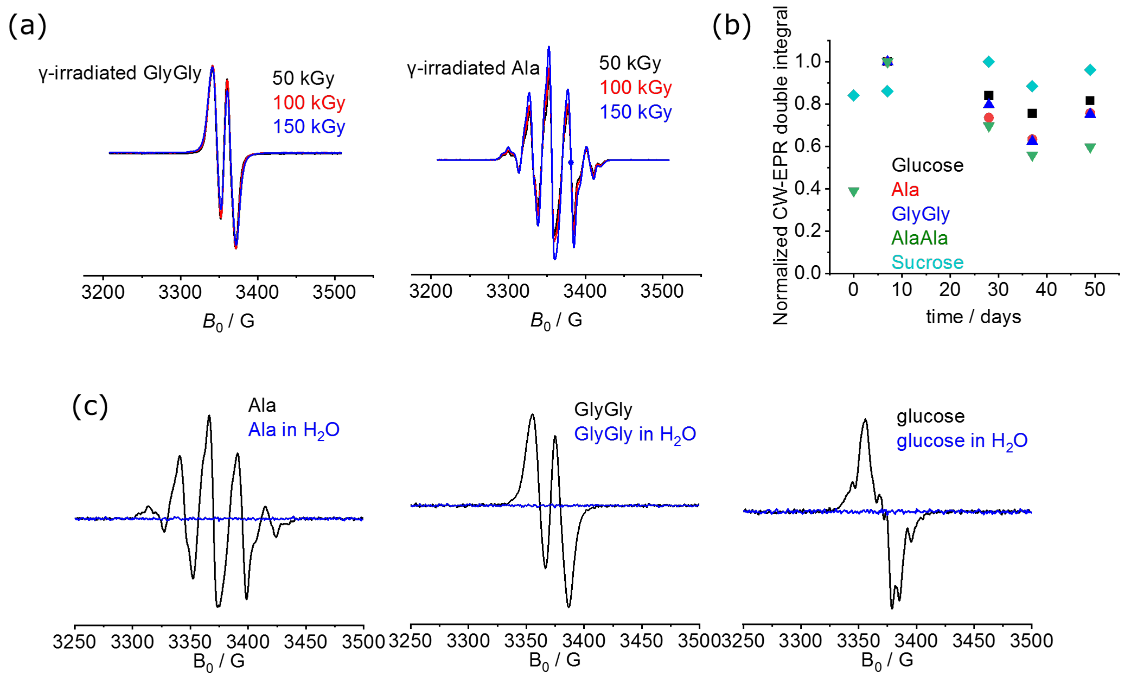

Figure S1. Room temperature X-band (a) CW-EPR spectra of 1 mg of two of the irradiated powders sealed under vacuum and irradiated with different doses of γ-irradiation, (b) double integrals of the CW-EPR spectra over time for some of the powders and (c) CW-EPR spectra of 1 mg γ-irradiated powders measured immediately after dissolving them in H_2O (blue) vs the spectrum of 1 mg powder (black) under same conditions. CW-EPR conditions: 25 dB in (a) or 30 dB in (c), conversion time 40 ms, modulation amplitude 1 G, modulation frequency 100 kHz, 1 scan.

2. Additional solid-state ^{1}H NMR data in the Hypersense® (HS) polarizer

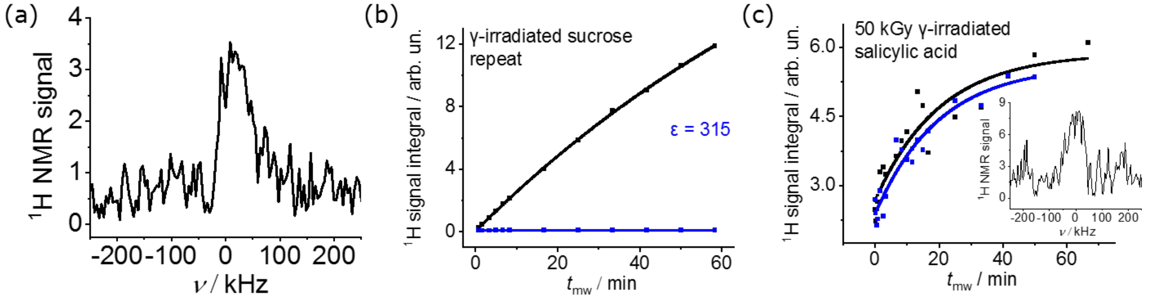

Figure S2. ^{1}H signals of empty cup at 1.5 K (a) and ^{1}H signal vs microwave irradiation times of γ-irradiated (b) sucrose from batch #1 and (c) salicylic acid from batch #2 at 1.5 K measured on the HS polarizer in presence and absence of microwaves (black and blue points, respectively) using a 4 µs pulse length, following a train of pre-saturation pulses (100 µs pulses, × 100). Microwave conditions: 94.05 GHz, 100 mW in (b) and150 mW power in (a, c). The microwave off data in (b) could not be fitted to a mono-exponential function (as in **Fig. 2d**): it fell on a straight line whose end value was taken as the "off" signal. The inset in (c) gives a representative solid-state ^{1}H NMR spectrum recorded with 4 µs pulse length and 100 s polarization time in presence of microwaves.

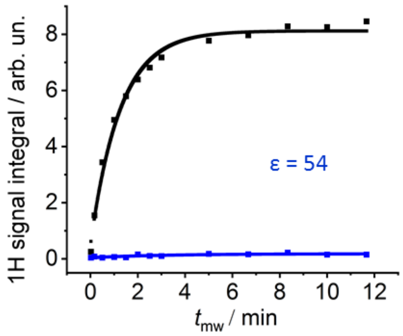

Figure S3. (a) ^{1}H signal vs microwave irradiation times of 10 mM 4-aminoTEMPO in H_2O/glycerol (3/2), recorded on the HS polarizer (93.99 GHz, 150 mW power) at 1.5 K and a home-built ^{1}H coil tuned at ~143 MHz. ^{1}H signals were measured in the presence and absence of microwaves (black and blue points, respectively) using a 20 µs pulse length, following a train of pre-saturation pulses (100 µs pulses, × 100). The solid black and blue lines are the fit to the data using a single exponentials, from which enhancement were calculated as described in the main text.

3. Frequency swept DNP profiles arising in the HS polarizer

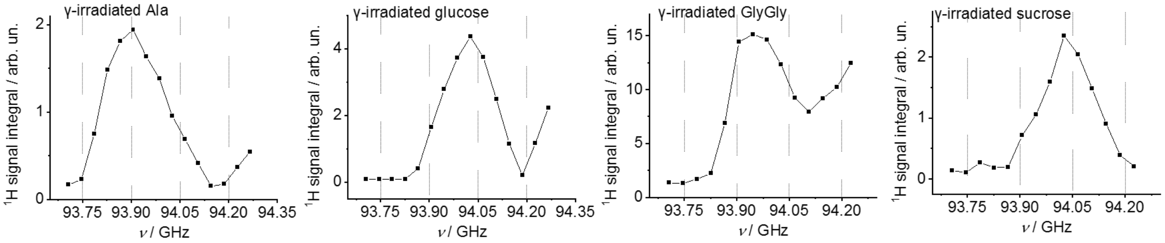

Figure S4. ^{1}H signals vs mw frequency on 150 kGy γ-irradiated powders (indicated samples) in the solid state measured on a HS polarizer at 1.5 K using a home-built ^{1}H coil tuned at ~143 MHz. ^{1}H signals were measured as described in Experimental, using mw irradiation times of 4 min each point and 150 mW of power.

4. Additional data on 150 kGy irradiated ATP

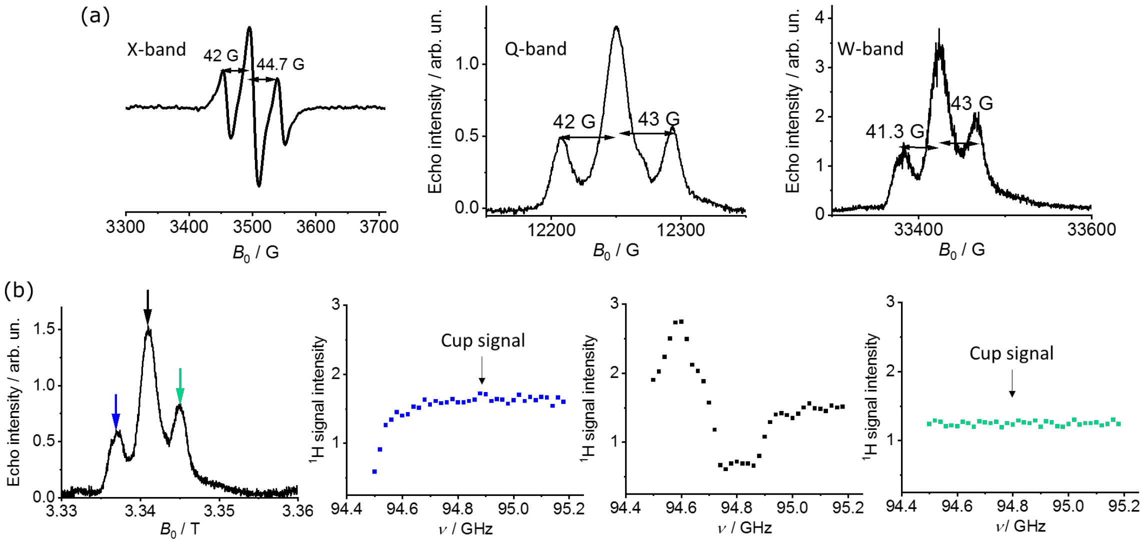

Figure S5. (a) Room temperature CW-EPR spectrum (left), room temperature Q-band ED-EPR spectrum (middle) and 5 K W-band ED-EPR (right) of 150 kGy irradiated ATP. In the spectra are denoted the values of the splitting of the EPR line which are found to be constant at three different magnetic fields. As the hyperfine coupling is independent of the field these data support the observed lineshape comes from hyperfine coupling of the ^{14}N nucleus to the radical of ATP. The Q-band ED-EPR spectrum was recorded with 90˚ – τ – 180˚ – τ – echo sequence with a 90˚ pulse of 14 μs, echo delay time τ = 400 ns repetition time 3 ms, ν= 34.1202 GHz, 49 scans. (b) DNP profile of irradiated ATP measured at different positions of the EPR spectrum (indicated on the left panel). When the DNP profile is measured on the left or right peaks (marked with blue and green arrow, respectively) a background signal with an amplitude of ~125 is recovered and can be assigned to the ^{1}H signal of the sample cup made of Teflon.

5. Additional solid-state ^{1}H NMR data on the hybrid NMR-EPR spectrometer

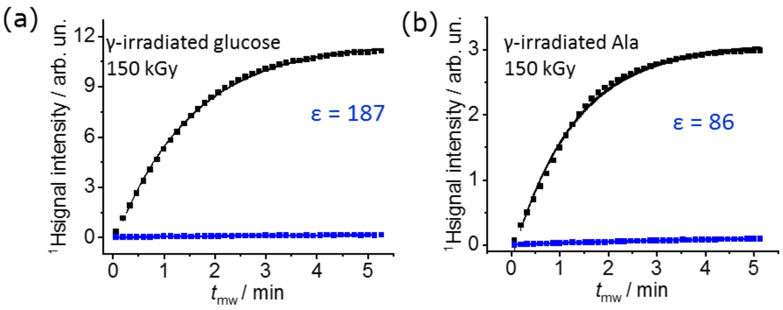

Figure S6. DNP results arising for 150 kGy γ-irradiated glucose from a batch different from that used in Fig. 3. ^{1}H signals vs buildup times were here measured at 94.98 GHz (glucose) or 94.68 GHz (Ala) using 4 μs (glucose) or 8 μs (Ala) 90˚ pulse in presence and absence of microwaves (black and blue points, respectively). Solid lines are fits to the data using a single exponential component and the reported enhancement calculated as described in the text.

6. Post-dissolution ^{1}H and ^{13}C NMR FID profiles

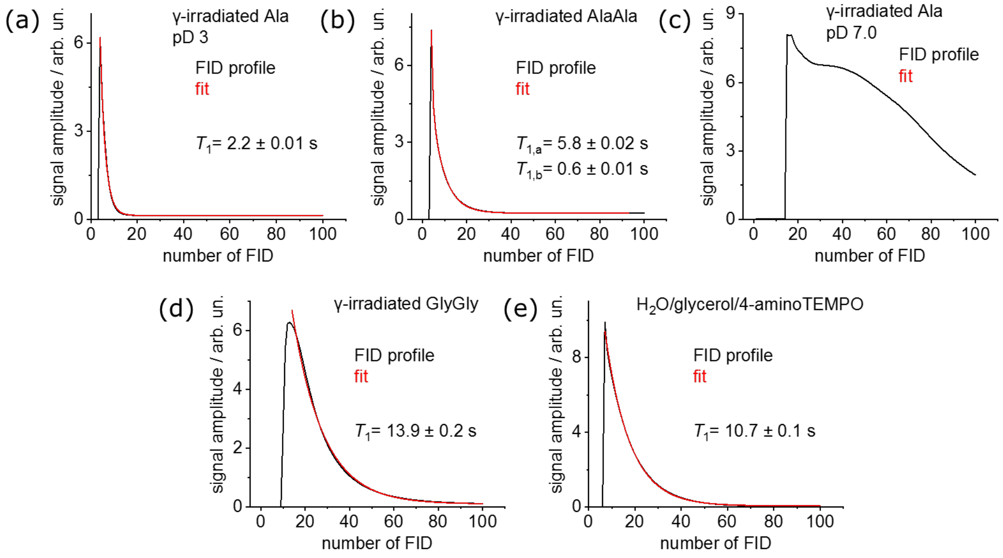

Figure S7. ^{1}H signal decay (single-point FID profiles, shown in black traces) observed upon utilizing 3.5 mL of D_2O to dissolve DNP-enhanced samples of γ-irradiated (a) Ala pD 3, (b) AlaAla pD 7, (c) Ala pD 7 and (d) GlyGly pD 7. Shown in (e) are results from water/glycerol co-mixed with 20 mM 4-aminoTEMPO. The data of Ala at pD 7 are affected by radiation damping. AlaAla was fitted by a bi-exponential function whereas the rest of data by a mono-exponential function (fits in red). NMR data recording began ca. 5 s prior to the sample's injections.

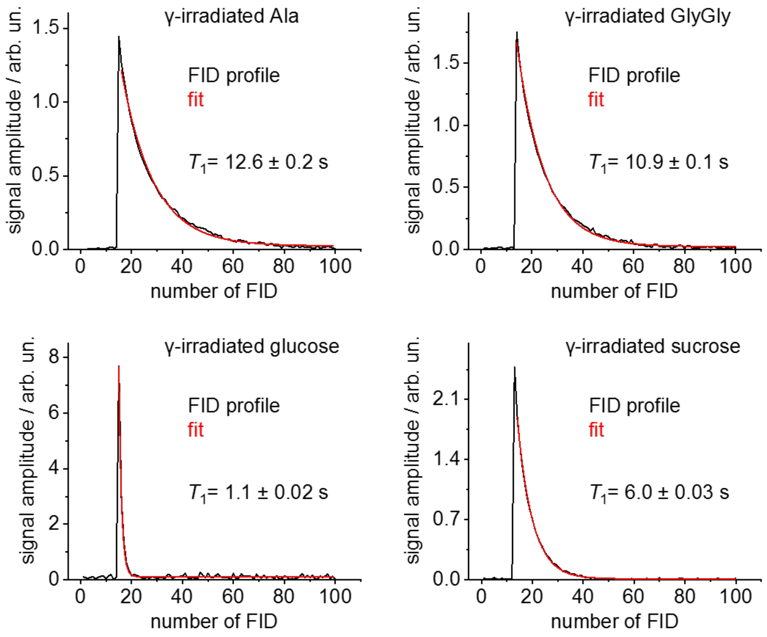

Figure S8. ^{13}C signal decay (single-point FID point profile) for the γ-irradiated powders indicated on each panel after dissolution with 3.5 mL H_2O/D_2O (9/1) (black). Fitting of the data to a mono-exponential decay function (red) gave the exponent which reflects ^{13}C T_1 relaxation. NMR data recording began ca. 10 s prior to the sample's injections.

7. DNP/NMR data on $^{13}C_1$-pyruvic/BDPA

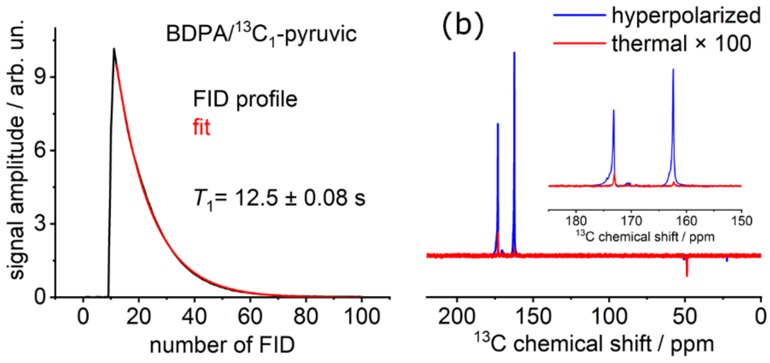

Figure S9. ^{13}C data on $^{13}C_1$-pyruvic/BDPA after dissolution. (a) ^{13}C signal decay (single-point FID point profile); fitting of the data to a mono-exponential decay function (red) gave the exponent reflecting the joint effects of the pulsing and ^{13}C T_1 relaxation. (b) Hyperpolarized and thermal ^{13}C solution NMR spectra with the inset focusing on the 150-190 ppm region. 40 µl of BDPA dissolved in sulfolane and co-mixed with $^{13}C_1$-pyruvic (1/1 v/v) were polarized on a HS polarizer at 1.5 K and at 94.040 GHz using 160 mW power, 2 hrs polarization time. Dissolution was done with 4 mL MeOH and spectra were recorded on a 500 MHz ^{1}H frequency spectrometer and data recording began ca. 10 s prior to the sample's injections. NMR parameters: 310 K, 1 s acquisition time, 0.05 s relaxation delay, 2 µs (≈13°) pulse length, 1 scan. The data were recorded as pseudo-2D with the time being the pseudo dimension and a time interval of 1 s from spectrum to spectrum. Hyperpolarized (immediately post-dissolution) vs thermal (FID at 100 s) spectra are shown in blue and red color, respectively. For calculating the enhancements, a thermal spectrum was recorded with 100 scans after the end of the dissolution process and upon sample reaching equilibrium.

8. DNP/NMR data on U-$^{13}C_6$-glucose after 150 kGy γ-irradiation

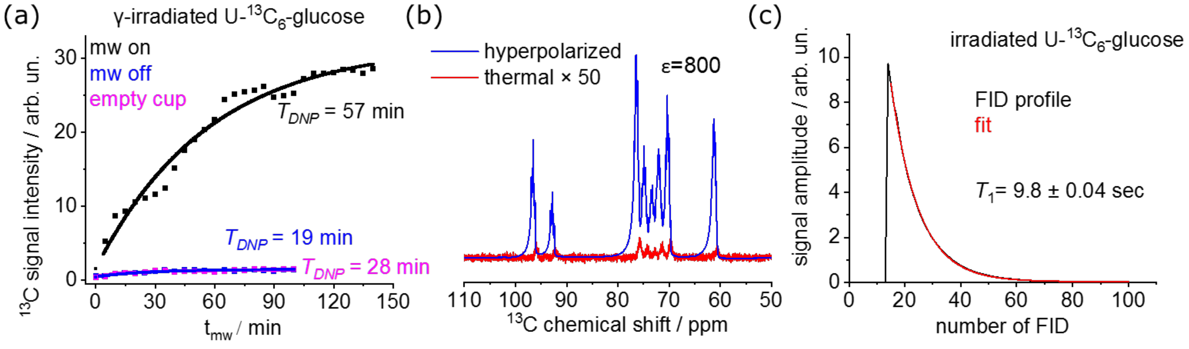

Figure S10. NMR/DNP ^{13}C data on U-$^{13}C_6$-glucose. (a) ^{13}C signals of 25 mg sample vs mw irradiation times in the solid state (~1.5 K) measured on a HS polarizer in presence and absence of microwaves (black and blue color, respectively) having as reference the corresponding measurement for an empty sample cup (magenta color). The solid lines are the fit to the data using a single exponential component from which the given buildup times were estimated. Microwave conditions: 160 mW, 94.106 GHz, 2 hrs polarization time. (b) Hyperpolarized and thermal ^{13}C solution NMR spectra. Dissolution was done with 4.0 mL H_2O/D_2O (9/1) and spectra were recorded on a 500 MHz ^{1}H frequency spectrometer at 330 K using 1 s acquisition time, 0.05 s relaxation delay, 2 µs (≈13°) pulse length, 1 scan. The data were recorded as a pseudo-2D experiment with the post-dissolution time being the "indirect" pseudo-dimension and a time interval of 1 s from spectrum to spectrum. Hyperpolarized (immediately post-dissolution) vs thermal (FID at 100 s)

spectra are shown in blue and red, respectively. For calculating the enhancement (of all peaks together), a thermal spectrum was recorded with 200 scans after the end of the dissolution process using a long relaxation delay. (c) Post-dissolution ^{13}C signal decay (single-point FID point profile). Fitting of the data to a mono-exponential decay function (red) gave the apparent T_1 exponent; ^{13}C NMR data recording began ca. 10 s prior to the sample's injections.

9. DNP/NMR data on U-$^{13}C_6$-glucose/Ox063

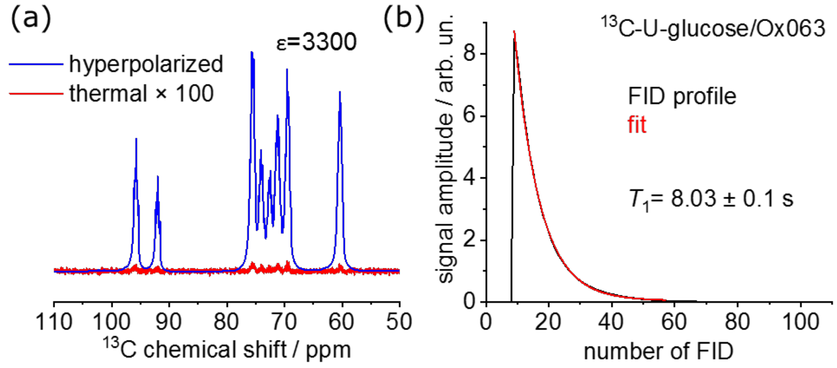

Figure S11. NMR/DNP ^{13}C data on 4 M U-$^{13}C_6$-glucose co-mixed with 15 mM Ox063 radical. 20 µL of sample were placed in a HS polarizer and were subjected to microwave irradiation (94.040 GHz) at ~1.5 K using 160 mW. Following a1.5 hr polarization time, dissolution was done with 4.0 mL phosphate buffer. (a) Hyperpolarized and thermal ^{13}C solution NMR spectra recorded on a 500 MHz ^{1}H frequency spectrometer at 330 K using 1 s acquisition time, 0.05 s relaxation delay, 2 µs (≈13˚) pulse length, 1 scan. The data were recorded as pseudo-2D with the time being the pseudo dimension and a time interval of 1 s from spectrum to spectrum. Hyperpolarized (immediately post-dissolution) vs thermal (FID at 100 s) spectra are shown in blue and red, respectively. For calculating the enhancement (of all peaks together), a thermal spectrum was recorded with 100 scans after the end of the dissolution process, with all scans reaching equilibrium. (b) ^{13}C signal decay (single-point FID point profile) after dissolution. Fitting of the data to a mono-exponential decay function (red) gave the exponent which reflects ^{13}C T_1 relaxation. NMR data recording began ca. 10 s prior to the sample's injections.

10. Summary of all samples used for DNP measurements

Table S1. Summary of the samples used for the solid-state and dissolution DNP experiments, indicating in which Figures each sample appears. Those marked with blue color denote samples prepared under the same conditions, but belonging to a different batch.

	**Irradiation dose / kGy**		
**compound**	**50**	**100**	**150**
Ala	Fig. 3, Fig. S1	Fig. S1	Fig. 2, Fig. 5, Fig. 6, Fig. S1, Fig. S4, Fig. S6, Fig. S7, Fig. S9
GlyGly	Fig. 3, Fig. S1	Fig. S1	Fig. 2, Fig. 4, Fig. 5, Fig. 6, Fig. S1, Fig. S4, Fig. S7, Fig. S9
glucose		Fig. 3	Fig. 2, Fig. 6, Fig. S1, Fig. S4, Fig. S6, Fig. S9
sucrose	Fig. 3		Fig 2, Fig. 4, Fig. 6, Fig. S2, Fig. S4, Fig. S9
AlaAla	Fig. 3, Fig. 5, Fig. S7		
AlaAsp	Fig. 1		

GlyAla	Fig. 1		
Gly methylester HCl	Fig. 1		
ATP	Fig. 1		Fig. 3, Fig. S6
succinic acid	Fig. 1		
sodium pyruvate	Fig. 1		
ibuprofen	Fig. 1		
salicylic acid	Fig. 1		Fig. S2
DL-4-fluorophenylAla	Fig. 1		
